# Identification of postsynaptic phosphatidylinositol-4,5-bisphosphate (PIP_2_) roles for synaptic plasticity using chemically induced dimerization

**DOI:** 10.1038/s41598-017-03520-3

**Published:** 2017-06-13

**Authors:** Su-Jeong Kim, Min-Jae Jeong, Hee-Jung Jo, Jung Hoon Jung, Bong-Kiun Kaang, Yun-Beom Choi, Joung-Hun Kim

**Affiliations:** 1 Department of Life Sciences, Pohang University of Science and Technology (POSTECH), Pohang, Gyungbuk 37673 Korea; 2 Department of Biological Sciences, College of Natural Sciences, Seoul National University, Seoul, 08826 Korea; 3 Neurology Service, VA New Jersey Health Care System, East Orange, New Jersey 07018 USA

## Abstract

Phosphatidylinositol-4,5-bisphosphate (PIP_2_), one of the key phospholipids, directly interacts with several membrane and cytosolic proteins at neuronal plasma membranes, leading to changes in neuronal properties including the feature and surface expression of ionotropic receptors. Although PIP_2_ is also concentrated at the dendritic spines, little is known about the direct physiological functions of PIP_2_ at postsynaptic as opposed to presynaptic sites. Most previous studies used genetic and pharmacological methods to modulate enzymes that alter PIP_2_ levels, making it difficult to delineate time- or region-specific roles of PIP_2_. We used chemically-induced dimerization to translocate inositol polyphosphate 5-phosphatase (Inp54p) to plasma membranes in the presence of rapamycin. Upon redistribution of Inp54p, long-term depression (LTD) induced by low-frequency stimulation was blocked in the mouse hippocampal CA3-CA1 pathway, but the catalytically-dead mutant did not affect LTD induction. Collectively, PIP_2_ is critically required for induction of LTD whereas translocation of Inp54p to plasma membranes has no effect on the intrinsic properties of the neurons, basal synaptic transmission, long-term potentiation or expression of LTD.

## Introduction

Although phosphatidylinositol-4,5-bisphosphate (PIP_2_) is a substrate for the generation of the second messengers inositol triphosphate (IP_3_) and diacylglycerol (DAG), PIP_2_ itself also interacts with membrane and cytosolic proteins to regulate a number of cellular processes in neurons. It is suggested that PIP_2_ directly controls the activity of ion channels and transporters^1^, which results in drastic changes in neuronal properties^2, 3^. For example, PIP_2_ binding regulates the activities of the KCNQ and inward-rectifying potassium channels (Kirs) that determine neuronal excitability^3–5^. Adaptor protein-2 (AP-2), which interacts with PIP_2_, is causally involved in the trafficking of synaptic vesicles and neurotransmitter receptors through clathrin-mediated endocytosis^6, 7^. Indeed, exocytosis and recycling of synaptic vesicles at presynaptic sites are affected by the amount of available PIP_2_^1, 8^. Depletion of PIP_2_ results in a smaller pool of readily releasable synaptic vesicles as well as delayed endocytosis and recycling^7^.

Despite the large volume of evidence for the actions of PIP_2_ at presynaptic sites, we have only limited knowledge on the physiological roles of PIP_2_ at postsynaptic sites, although PIP_2_ is also concentrated at the plasma membrane of dendrites^9^. At postsynaptic sites, endocytosis of membrane proteins maintains surface expression of N-methyl-D-aspartate receptors (NMDARs) and α-amino-3-hydroxy-5-methyl-4-isoxazolepropionic acid receptors (AMPARs)^10^. It was previously shown that PIP_2_ facilitates the surface expression of NMDARs in cultured cortical neurons while loss of PIP_2_ enhances clathrin-dependent NMDAR internalization by promoting cofilin depolymerization of the actin cytoskeleton^11^. Internalization of AMPAR by cultured hippocampal neurons is blocked by a lack of synaptojanin that mediates PIP_2_ dephosphorylation^12^.

Genetic modification of enzymes that alter PIP_2_ levels affects properties related to NMDAR-dependent synaptic plasticity, such as long-term depression (LTD)^13–15^. For example, deletion of phosphatidylinositol 3-kinase γ (PI3Kγ) which phosphorylates PIP_2_ to generate PIP_3_, impairs LTD^16^. However, such previous studies reported conflicting results and thus do not provide unequivocal evidence supporting whether and how PIP_2_ controls synaptic plasticity^14–16^. This ambiguity is likely due to methodological differences among different studies, as modulation of PIP_2_ levels was achieved by genetic and pharmacological modifications of PIP_2_-metabolic enzymes, such as synaptojanin 1, phosphatase and tensin homolog (PTEN), phospholipase C (PLC), and phosphatidylinositol 4-phosphate 5-kinases (PIP5Ks). Because these enzymes affect other proteins as well as control PIP_2_ levels^17–19^, the possibility cannot be excluded that the findings of previous studies resulted from unintended effects of PIP_2_-metabolic enzymes on other signaling molecules rather than on PIP_2_. In addition, genetic modification can lead to developmental compensatory effects and a number of potentially non-physiological outcomes towing to the protracted time courses of the modification. Although pharmacological approaches allow for elucidation of time-specific effects, it is almost impossible to distinguish between the roles of PIP_2_ in pre- or post-synapses because pharmacological agents diffuse throughout brain tissue.

To resolve the discrepancy and to obtain better insight into the direct effects of PIP_2_ on LTD, we developed a means to acutely deplete PIP_2_ in hippocampal neurons using chemically-induced dimerization (CID), which utilized the heterodimerization of the domain from the FK506-binding protein (FKBP) and the FKBP rapamycin-binding (FRB) domain from the mechanistic target of rapamycin (mTOR). A PIP_2_-specific phosphatase, inositol polyphosphate 5-phosphatase (Inp54p), was translocated to the plasma membrane of neurons in the presence of rapamycin and promptly depleted PIP_2_, as previously shown in other cell types^20–23^. Using this CID system, we then determined whether PIP_2_ controls synaptic transmission and plasticity in the Schaffer collateral-CA1 pathway in mouse hippocampus. We also elucidated the time frame during which PIP_2_ is required for NMDAR-dependent LTD. Our results indicated that PIP_2_ is necessary for the induction of LTD but not for LTD expression or synaptic transmission.

## Results

### CID induces acute reduction in membrane PIP_2_ levels

**Figure 1:**
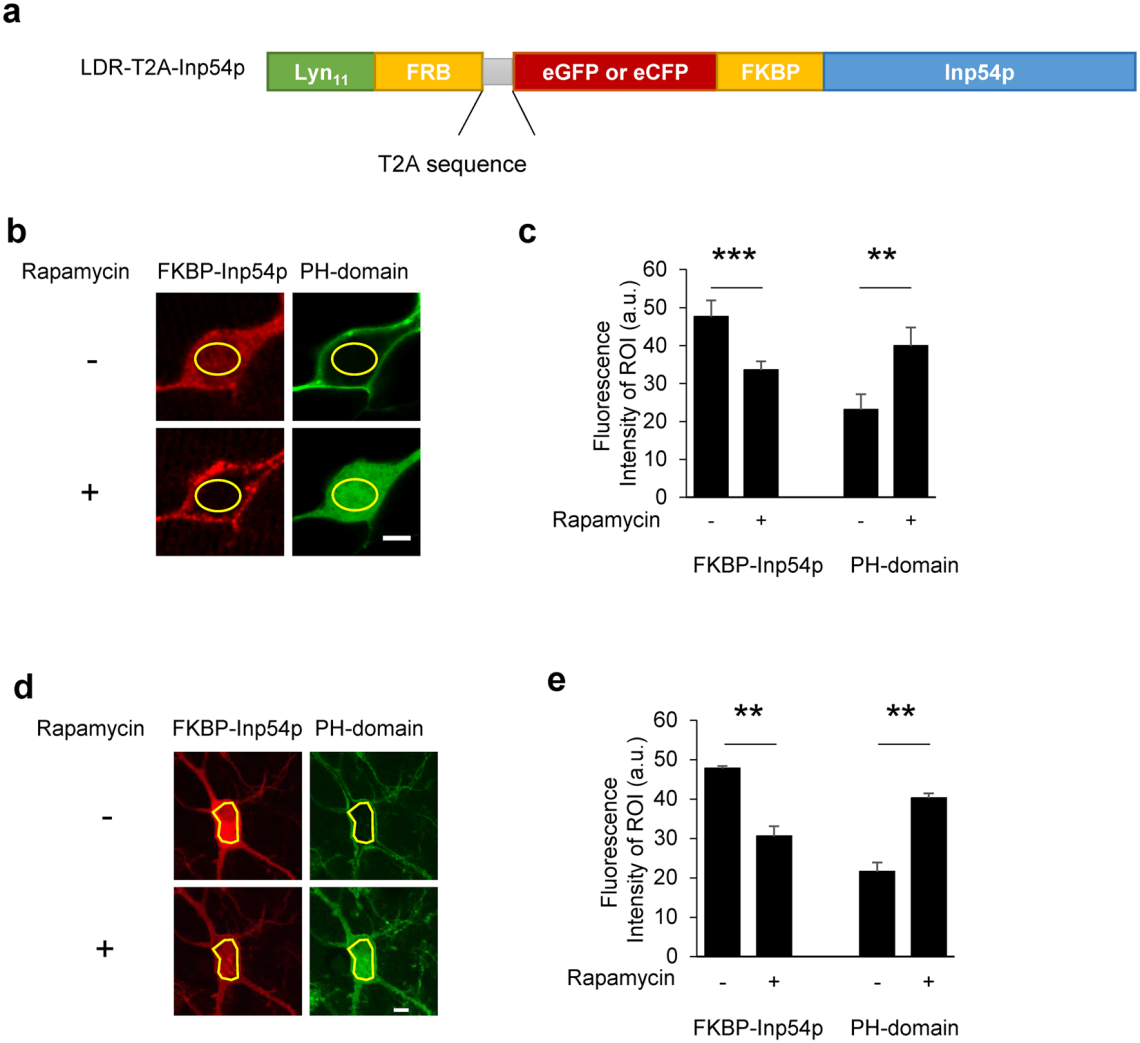
Validation of chemically induced dimerization for PIP_2_ depletion using a single plasmid, LDR-T2A-Inp54p. (**a**) Schematic representation of the construct of the plasma membrane PIP_2_ depletion system using a viral T2A peptide sequence linker. (**b**) Representative images of a HEK293T cell expressing LDR, FKBP-Inp54p (red), and PH-domain (green) before (−) and after (+) treatment with 100 nM rapamycin. Areas outlined with yellow lines represent the cytoplasmic region of interest (ROI). Scale bars: 5 μm. (**c**) Mean fluorescence intensity of ROI in cells expressing LDR, FKBP-Inp54p, and PH-domain before and after treatment with 100 nM rapamycin in HEK293T cells (FKBP-Inp54p: 47.63 ± 4.30 *vs*. 33.53 ± 2.32, ***p < 0.001; PH-domain: 23.11 ± 4.09 *vs*. 39.92 ± 4.88, **p < 0.01, N = 4, n = 20, Wilcoxon test). (**d**) A representative neuron expressing LDR, FKBP-Inp54p (red), and PH-domain (green) before (−) and after (+) 100 nM rapamycin treatment. Areas outlined with yellow lines represent the cytoplasmic region of interest (ROI). Scale bars: 10 μm. (**e**) Mean fluorescence intensity of ROI in hippocampal neurons expressing LDR, FKBP-Inp54p, and PH-domain before and after 100 nM rapamycin treatment (FKBP-Inp54p: 47.80 ± 0.60 vs. 30.55 ± 2.57, **p < 0.01; PH-domain: 21.54 ± 2.37 vs. 40.23 ± 1.26, **p < 0.01, N = 2, n = 6, Wilcoxon test).

To elucidate the physiological roles of PIP_2_ at postsynaptic neurons by precisely modulating membrane PIP_2_ levels of live neurons in hippocampal slices, we adapted a CID system that has been previously used mainly for non-neuronal cell types^21, 22^. For *in situ* CID, cells need to express two components, membrane-targeted proteins and PIP_2_-specific phosphatases, that allow rapid metabolism of PIP_2_ within the plasma membrane^22^. Although this CID system has been used in several cell types, it would be difficult to introduce two components at an equimolar ratio into individual neurons and extend this system to brain slices and even intact animals^24, 25^. Thus, we designed a new viral construct using the 2 A peptide sequence from the insect *Thosea asigna* virus (T2A) to link the two components^26^: The enhanced cyan fluorescent protein (eCFP) or the enhanced green fluorescent protein (eGFP) sequence fused to the N-terminus of FKBP-Inp54p (FKBP-Inp54p) was linked to Lyn_11_-FRB (LDR), a membrane-anchored FRB, via a T2A-linked sequence (LDR-T2A-Inp54p) (Fig. 1a).

We examined whether the application of rapamycin could lead to recruitment of Inp54p to the plasma membrane and subsequently reduce of the membrane PIP_2_ level. Live-cell imaging of HEK293T cells revealed that FKBP-Inp54p normally remained in the cytosolic space but moved to the plasma membrane within 3 min of rapamycin treatment (Fig. 1b,c). We also co-transfected individual HEK293T cells with the pleckstrin homology domain of PLC-δ, PIP_2_-binding domain tagged with either eGFP or mCherry (PH-domain)^27^, and thereby tested whether the membrane-targeted Inp54p was able to metabolize PIP_2_. As expected, rapamycin treatment resulted in translocation of the PH-domain from the plasma membrane to the cytosol, indicating that membrane-bound PIP_2_ was readily depleted by Inp54p within 3 min. We next examined whether the CID moved to the cell membrane and subsequently reduced membrane PIP_2_ levels in the hippocampal neurons. Prior to rapamycin treatment, FKBP-Inp54p was widely distributed in soma and neurites of neurons, while the PH-domains were largely located in the plasma membrane. Rapamycin treatment caused FKBP-Inp54p to translocate from cytosolic areas to the plasma membrane. The PH-domains that were present in soma and neurites were released into the cytosolic compartment. These results indicated that PIP_2_ is rapidly metabolize by FKBP-Inp54p and rapamycin treatment (Fig. 1d,e).

**Figure 2:**
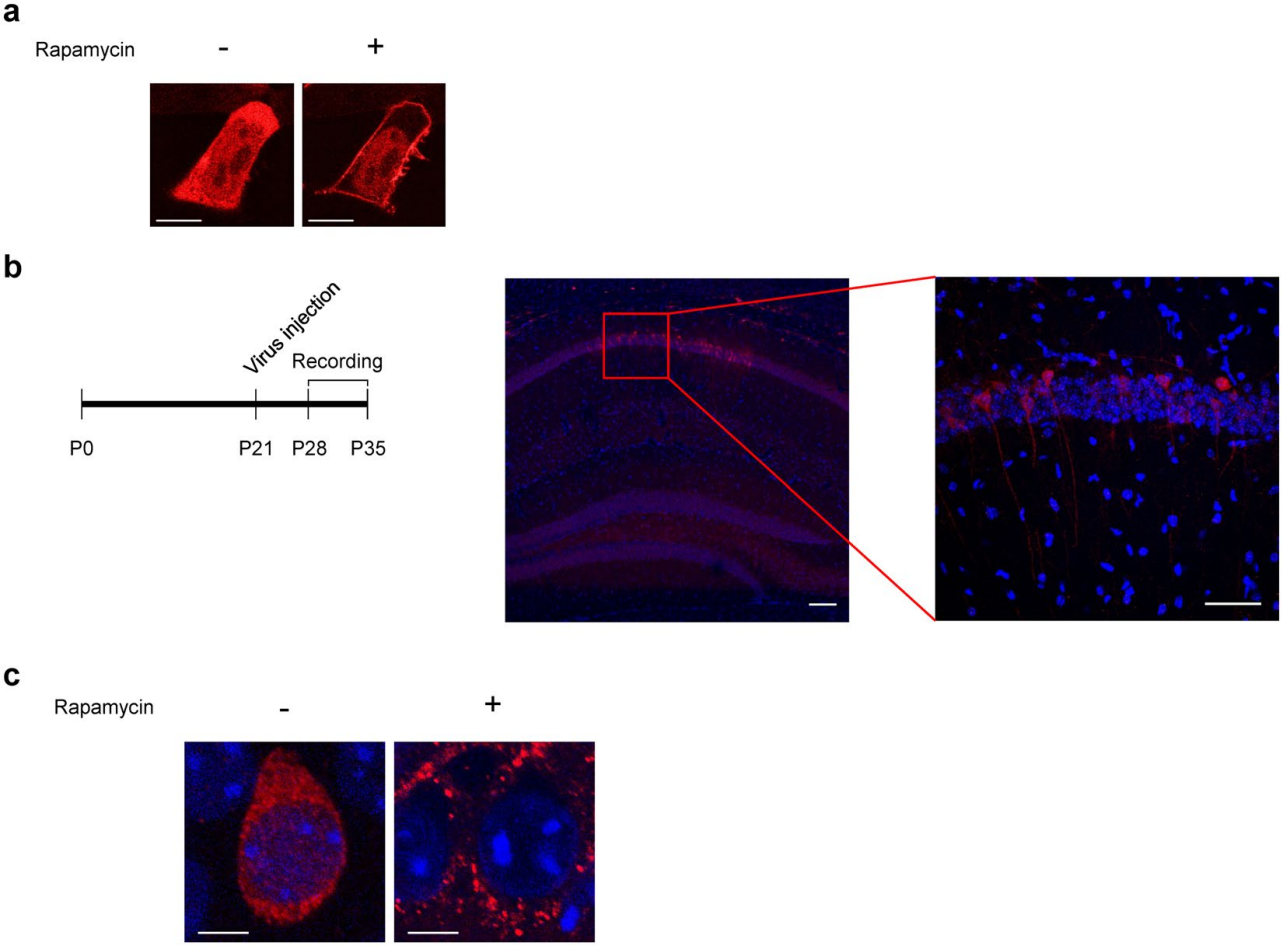
Viral expression of LDR-T2A-Inp54p in HEK293T cells and a mouse brain. (**a**) Example images of a HEK293T cell infected with lentivirus containing the chemically induced PIP_2_ depletion system before and after treatment with 100 nM rapamycin. Scale bar: 10 μm. (**b**) Experimental timeline for virus infusion and electrophysiological analysis (left). Representative images of lentiviral LDR-T2A-Inp54p expression in the mouse hippocampus showing DAPI (blue) and LDR-T2A-Inp54p expression (red) (middle). The magnified image shows the red rectangular area in the middle image (right). Scale bars: 100 μm (middle) and 50 μm (right). (**c**) Translocation of FKBP-Inp54p in brain slices with (+) and without (−) bath application of 100 nM rapamycin. FKBP-Inp54p (red), DAPI (blue) are shown. Scale bars: 5 μm.

To implement the CID system in intact neural circuits, we sought to produce viruses expressing LDR-T2A-Inp54p. Because the insert size ( > 5 kb) surpassed the packaging limit of adeno-associated viruses, we attempted to express LDR-T2A-Inp54p using a lentivirus that allows for long-term expression as well as for packaging of long genomes^28^. We produced a lentivirus containing LDR-T2A-Inp54p by co-transfecting LDR-T2A-Inp54p, VSVg, gag and pol constructs into HEK293T cells. The harvested virus successfully infected HEK293T cells (Fig. 2a). This virally expressed LDR-T2A-Inp54p also translocated from the cytosol to the plasma membrane within 3 min of rapamycin treatment, validating the effectiveness of our LDR-T2A-Inp54p virus (Fig. 2a).

Neural circuits in the hippocampal CA1 undergo extensive synaptic plasticity, which is subject to a number of neuronal modifications, such as trafficking of synaptic vesicles at presynaptic sites and transmitter receptors at postsynaptic sites^29–32^. Thus, pyramidal neurons innervated by the Schaffer collateral pathway are well-suited to examining the direct roles of PIP_2_ in synaptic plasticity. We infused the lentivirus into the CA1 region of the hippocampus in live mice on postnatal day 21 and waited for 7 to 10 days for full expression of LDR-T2A-Inp54p^33, 34^. As revealed by fluorescent eCFP signals, Inp54p was expressed selectively in the cell bodies and dendrites of CA1 pyramidal neurons (Fig. 2b). We also monitored the movement of FKBP-Inp54p after rapamycin treatment in hippocampal slices. In agreement with our *in vitro* data (Fig. 2a), bath application of rapamycin resulted in decreased expression of FKBP-Inp54p in cytosolic spaces (Fig. 2c). These results support the notion that treatment of rapamycin could induce the translocation of Inp54p in brain tissues as well as in cultured cells.

### CID-induced manipulation of PIP_2_ has no effect on intrinsic properties or synaptic transmission in pyramidal neurons

**Figure 3:**
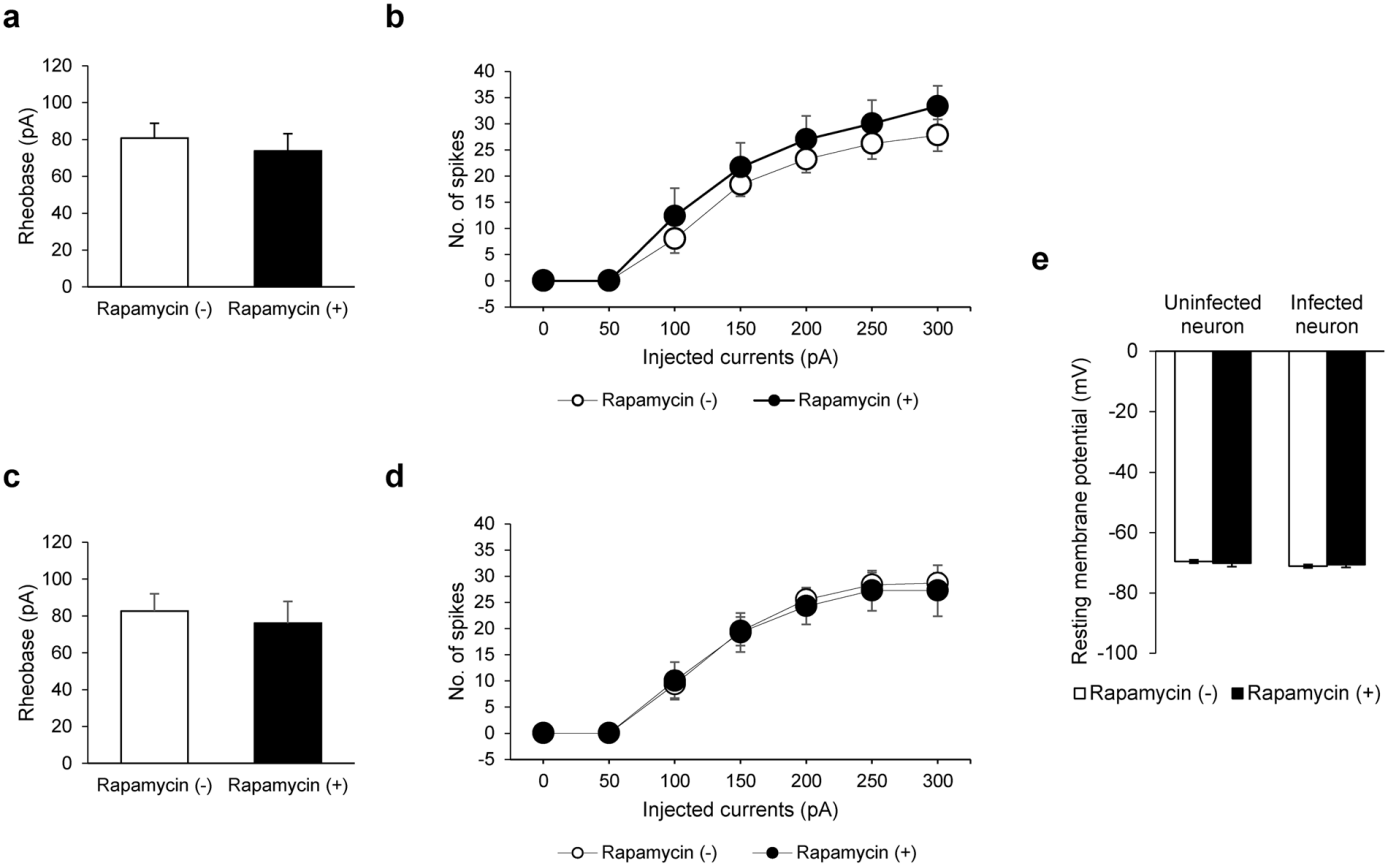
Lack of effect of rapamycin or acute reduction of PIP_2_ on neuronal excitability. (**a**) Mean rheobases in uninfected CA1 neurons with and without rapamycin (Rapamycin (−), 80.84 ± 8.02 pA, N = 4, n = 5 *vs*. Rapamycin (+), 73.80 ± 9.32 pA, N = 3, n = 6, p > 0.1, Mann-Whitney U-test). (**b**) The number of spike firings at the indicated current steps in uninfected CA1 neurons. (**c**) Mean rheobases in CA1 neurons expressing LDR-T2A-Inp54p with and without rapamycin. (Rapamycin (−), 82.67 ± 9.32 pA, N = 2, n = 5 *vs*. Rapamycin (+), 76.05 ± 11.8 pA, N = 2, n = 4, p > 0.1, Mann-Whitney U-test) (**d**) The number of spike firings at the indicated current steps in CA1 neurons expressing LDR-T2A-Inp54p. (**e**) Resting membrane potentials (RMPs) of hippocampal neurons before and after rapamycin treatment (Uninfected neurons: Rapamycin (−) −69.55 ± 0.55 mV *vs*. Rapamycin (+) −70.17 ± 1.12 mV, N = 3, n = 10, p > 0.1; Infected neurons: Rapamycin (−) −71.11 ± 0.59 mV *vs*. Rapamycin (+) −70.59 ± 0.97 mV, N = 3, n = 9, p > 0.1, Paired T-test). Error bars show mean ± SEM.

PIP_2_ binds to ion channels, including the KCNQ family and Kirs, and thereby regulates neuronal activities^35, 36^. If this is also the case in neurons, the CID-induced manipulation of PIP_2_ would potentially affect the intrinsic properties of hippocampal CA1 pyramidal neurons. To examine this possibility we first showed that rheobase, the smallest current amplitudes required to elicit a single spike (Fig. 3a), and the number of spikes elicited upon injection of current (Fig. 3b) remained unaltered before and after addition of rapamycin to uninfected CA1 pyramidal neurons, suggesting that rapamycin itself did not change intrinsic properties, such as membrane excitability, in CA1 pyramidal neurons. Then, we examined the intrinsic properties of CA1 neurons infected with lentivirus containing LDR-T2A-Inp54p. Because the level of membrane PIP_2_ did not decrease in the presence of Inp54p alone without rapamycin (Fig. 1), we examined whether translocation of FKBP-Inp54p by rapamycin could affect membrane excitability. The administration of rapamycin did not alter rheobase or the number of spikes following the injection of current, at least within the time window that we monitored in the current study (Fig. 3c,d). It is conceivable that the resting membrane potential (RMP) would be altered by our manipulation because a number of the ion channels that maintain the RMP are controlled by PIP_2_^37^. Inconsistent with this possibility, rapamycin-triggered translocation of FKBP-Inp54p did not affect the RMP in LDR-T2A-Inp54p infected neurons (Fig. 3e). Thus, the intrinsic properties of hippocampal neurons are largely indifferent to dynamic ranges of PIP_2_.

**Figure 4:**
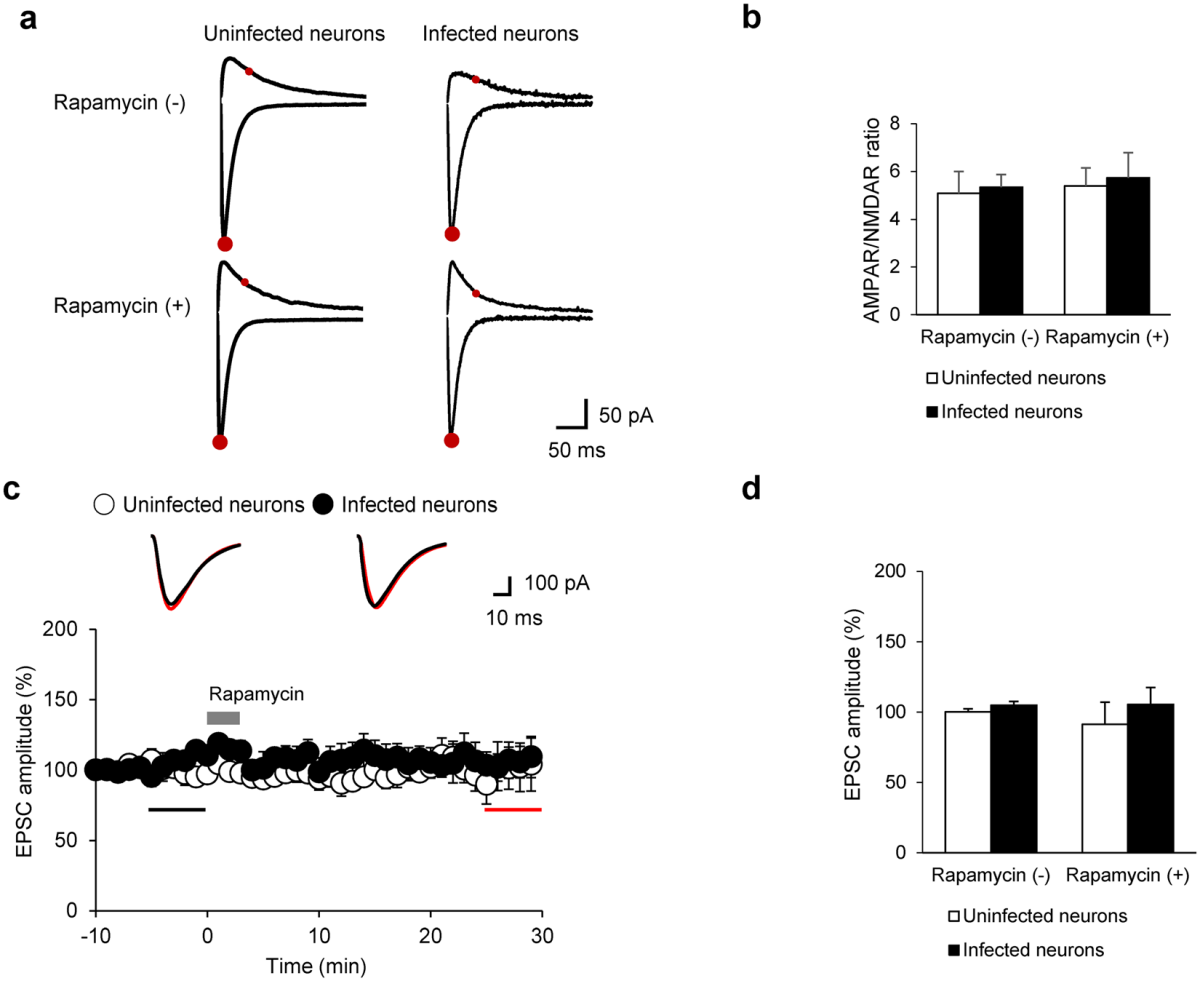
Lack of effect of rapamycin or acute reduction of PIP_2_ on excitatory synaptic transmission. (**a**) Representative traces of evoked EPSCs (−70 mV holding potential for AMPAR-EPSCs and +40 mV holding potential for NMDAR-EPSCs). AMPAR- (lower large red circles) and NMDAR-dependent EPSCs (upper small red circles) were measured. (**b**) Mean AMPAR/NMDAR ratios for each group (Rapamycin (−): uninfected 5.08 ± 0.92, N = 7, n = 11 *vs*. infected 5.34 ± 0.76, N = 5, n = 7, p > 0.1; Rapamycin (+): uninfected 5.39 ± 0.53, N = 6, n = 12 *vs*. infected 5.73 ± 1.06, N = 5, n = 6, p > 0.1, Mann-Whitney U-test). (**c**) Time course of the normalized amplitudes of EPSCs in hippocampus CA1 neurons. Amplitudes of EPSCs are normalized to baseline levels that were recorded for 10 min before treatment with rapamycin (gray bar). Inserts: representative traces with color- matched time points. (**d**) Mean EPSC amplitudes in CA1 neurons for each group. (Rapamycin (−): uninfected 100.23 ± 2.17%, N = 4, n = 5 *vs*. infected 104.93 ± 2.58%, N = 4, n = 4, p > 0.1; Rapamycin (+): uninfected 91.28 ± 15.68%, N = 4, n = 5 *vs*. infected 105.42 ± 12.13%, N = 4, n = 4, p > 0.1, Mann-Whitney U-test). Error bars show mean ± SEM.

Given the previous evidence indicating that the abundance of glutamate receptors in the plasma membrane could be differentially modulated by altered PIP_2_ levels^11, 14^, we elicited excitatory postsynaptic currents (EPSCs) in CA1 neurons while stimulating the Schaffer collateral pathway of hippocampal slices prepared from mice that had been infused with lentivirus containing LDR-T2A-Inp54p. Subsequently, we measured AMPAR-mediated and NMDAR-mediated EPSCs as previously described^38^ (Fig. 4a) and calculated the ratios of AMPAR- to NMDAR-EPSCs (A/N ratio), which is indicative of the nature of the synaptic transmission^9, 11, 14^. We did not observe any significant rapamycin-induced alteration in the A/N ratio of the CA1 pyramidal neurons infected with lentivirus containing LDR-T2A-Inp54p, and the A/N ratio in the infected neurons was comparable to the values obtained from the uninfected neurons (Fig. 4a,b). The observed absence of effect on synaptic transmission was surprising, given the previous reports suggesting that PIP_2_ could control the activity of either NMDARs or AMPARs^11, 39^. Because the constant A/N ratio might have resulted from concurrent shifts in AMPAR- and NMDAR-EPSCs, we continuously monitored the AMPAR-EPSCs that were recorded at a holding potential of –70 mV but failed to observe any change in AMPAR-EPSC amplitudes despite rapamycin treatment (Fig. 4c,d). Thus, synaptic transmission is unlikely to be affected by the PIP_2_ dephosphorylation *per se*. Interestingly, our results are inconsistent with those observed in previous studies based on relatively long-term manipulations of PIP_2_-regulating enzymes^14^.

### LTD induction affected by CID translocation

**Figure 5:**
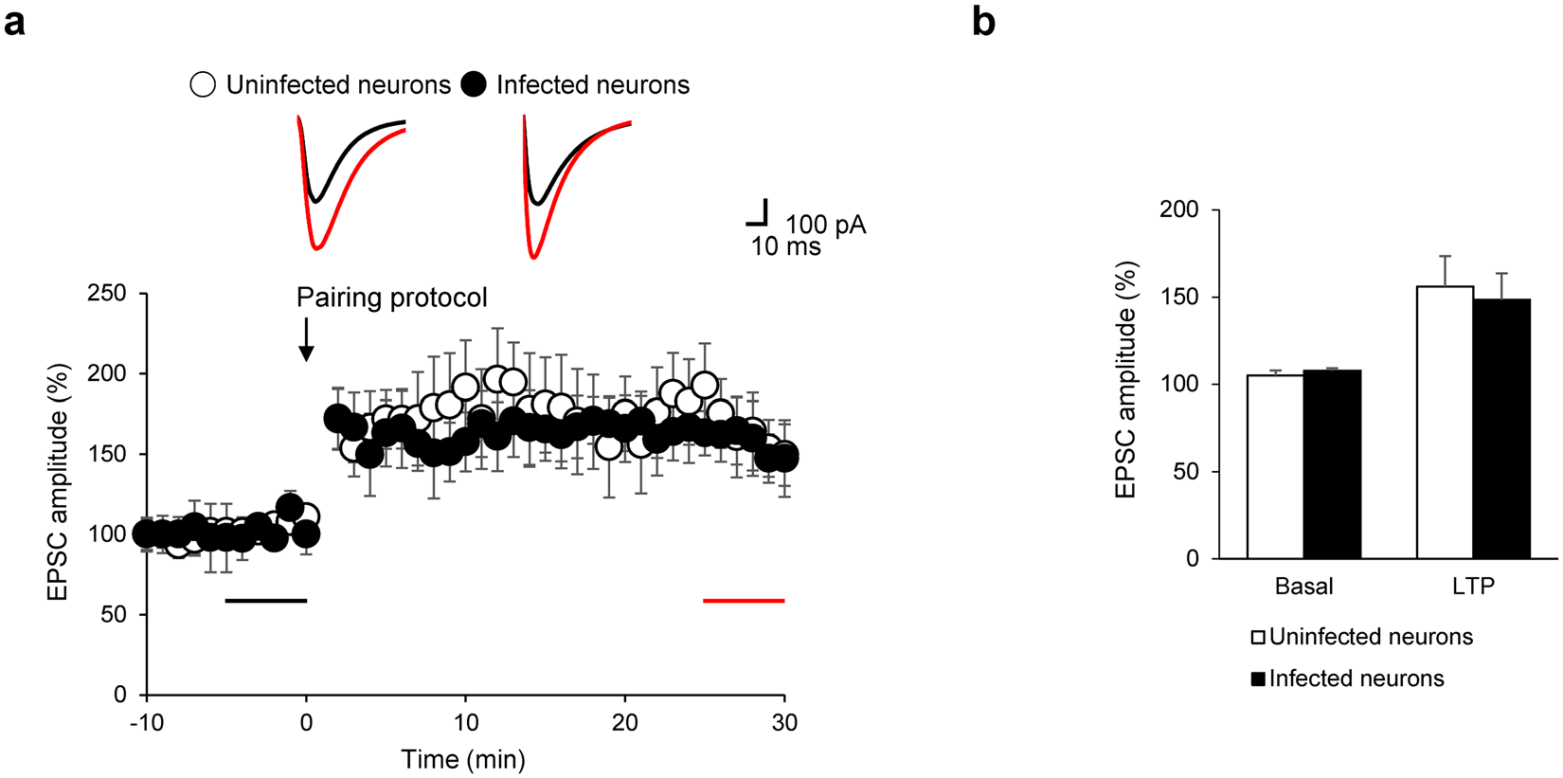
Induction and expression of pairing protocol-induced LTP were not affected by rapamycin or acute reduction of PIP_2_. (**a**) Normalized EPSC amplitudes before and after LTP induction. Amplitudes of EPSCs are normalized to baseline levels that were recorded for 10 min before the pairing protocol (arrow). Rapamycin treatment occurred prior to the beginning of the recording. Inserts: representative traces of evoked EPSCs with color- matched time points (**b**) Mean EPSC amplitudes before and after LTP induction in CA1 neurons for each group. (Basal: uninfected 105.09 ± 2.76%, N = 5, n = 5 *vs*. infected 107.83 ± 1.31%, N = 4, n = 4, p > 0.1, LTP: uninfected 156.16 ± 17.48%, N = 5, n = 5 *vs*. infected 148.60 ± 14.98%, N = 4, n = 4, p > 0.1, Mann-Whitney U-test). Error bars show mean ± SEM.

LTP impairment in aged animals has been attributed, at least in part, to reduced levels of PIP_2_^40^. To address this possibility, we examined whether acute depletion of PIP_2_ impaired synaptic plasticity, as has been shown in aged mice. We first assessed the effect of rapamycin on LTP at the concentration we had used for dephosphorylation of PIP_2_, as LTP can be abolished in the presence of rapamycin, likely via inhibition of mTOR signaling^41^. We applied rapamycin (100 nM) 3 min before the start of baseline recording, but did not detect a significant difference in induction or expression of LTP induced by pairing presynaptic stimulation (2 Hz, 80 pulses) with postsynaptic depolarization (0 mV) (Fig. 5a). Importantly, the magnitude of LTP in the neurons infected with the lentivirus containing LDR-T2A-Inp54p was comparable to that in uninfected neurons, indicating that rapamycin treatment and the resultant dephosphorylation of PIP_2_ via CID had no significant impact on LTP (Fig. 5b). Thus, induction and maintenance of LTP does not causally rely on normal levels of PIP_2_, at least in the CA1 pyramidal neurons, while chronic reduction of PIP_2_ levels can result in impairment of LTP^40^.

**Figure 6:**
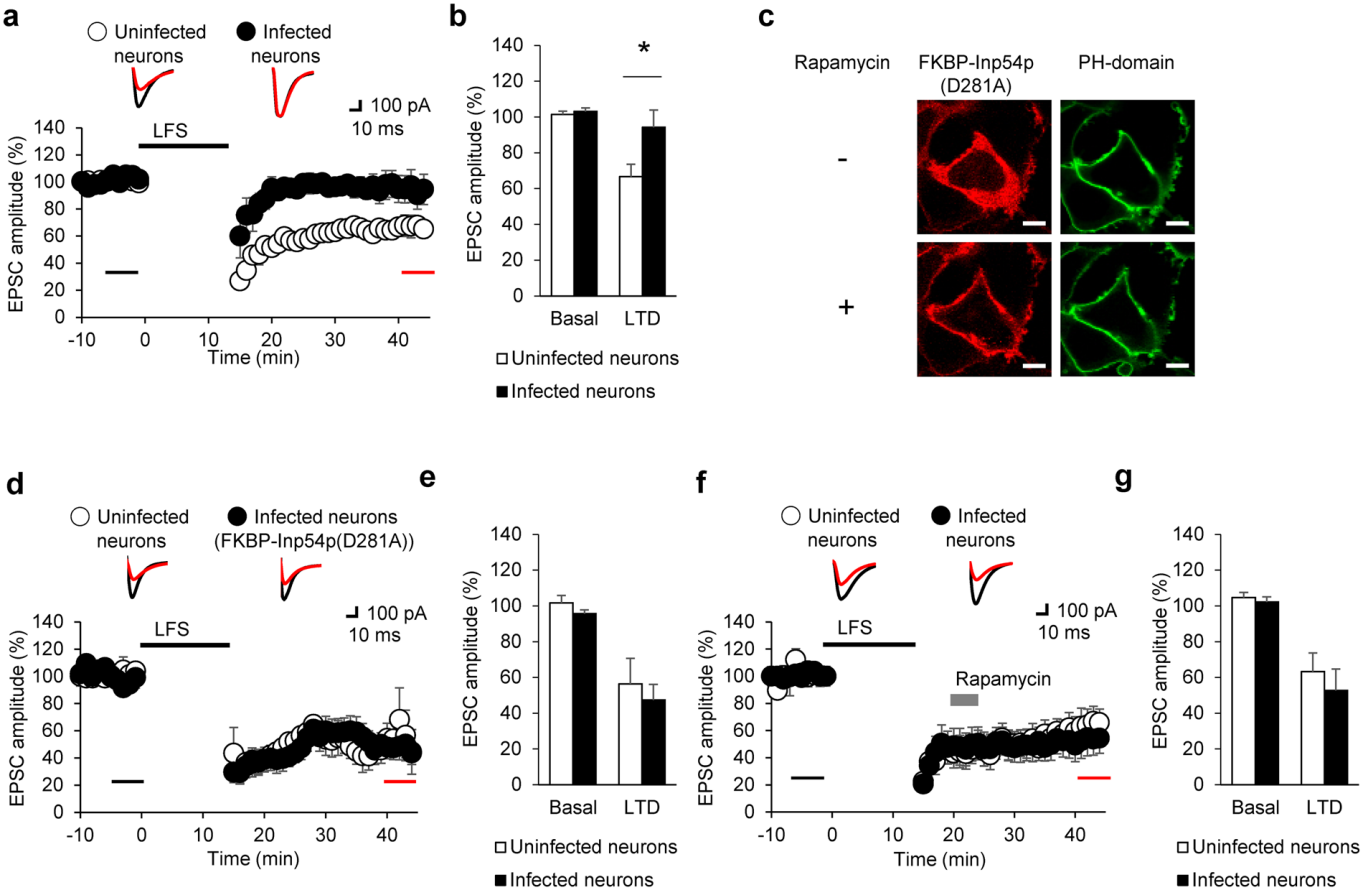
Acute PIP_2_ reduction disrupted induction but not expression of LTD. (**a**) Normalized EPSC amplitudes when PIP_2_ depletion occurred before the LTD induction. Rapamycin treatment was made prior to the beginning of the recording. Inserts: representative traces with color- matched time points. (**b**) Mean EPSC amplitudes before and after LTD induction in CA1 neurons for each group (Rapamycin (−): uninfected 101.44 ± 1.84%, N = 7, n = 9 *vs*. infected 103.15 ± 1.87%, N = 5, n = 5, p > 0.1; Rapamycin (+): uninfected 66.62 ± 6.91%, N = 7, n = 9 *vs*. infected 94.22 ± 9.62%, N = 5, n = 5, *p < 0.05, Mann-Whitney U-test). (**c**) Representative images of a HEK293T cell expressing LDR, FKBP-Inp54p(D281A) (red), and PH-domain (green) before and after 100 nM rapamycin treatment. Scale bars: 5 μm. (**d**) Normalized EPSC amplitudes from FKBP-Inp54p(D281A)-expressing neurons. Rapamycin treatment was made prior to the beginning of the recording. Inserts: representative traces with color- matched time points. (**e**) Mean EPSC amplitudes before and after LTD induction in CA1 neurons for each group (Rapamycin (−): uninfected 101.73 ± 4.10%, N = 4, n = 4 *vs*. infected 95.64 ± 2.18%, N = 3, n = 5, p > 0.1; Rapamycin (+): uninfected 56.40 ± 14.27%, N = 4, n = 4 *vs*. infected 47.42 ± 8.68%, N = 3, n = 5, p > 0.1, Mann-Whitney U-test). (**f**) Normalized EPSC amplitudes when PIP_2_ depletion occurred during the LTD expression. Rapamycin treatment occurred during LTD expression (gray bar). Inserts: representative traces with color- matched time points. (**g**) Mean EPSC amplitudes before and after the LTD induction in CA1 neurons for each group (Rapamycin (−): uninfected 104.61 ± 2.89%, N = 5, n = 6 *vs*. infected 102.22 ± 2.79%, N = 7, n = 7, P > 0.1; Rapamycin (+): uninfected 63.24 ± 10.47% N = 5, n = 6 *vs*. infected 52.78 ± 11.88%, N = 7, n = 7, p > 0.1, Mann-Whitney U-test). Error bars show mean ± SEM. All amplitudes of EPSCs are normalized to basal recording for 10 min before LTD induction by LFS (upper black bar).

PIP_2_ is an important factor in controlling NMDAR-dependent LTD in the hippocampus. For example, inhibition of PTEN, which dephosphorylates PIP_3_ to generate PIP_2_, interferes with LTD induction^14^. Moreover, the activity of PIP5Kγ661, the major PIP_2_-producing enzyme in the brain, is required for the endocytosis of AMPA receptors during LTD^15^. However, previous studies were unable to provide sufficient evidence to indicate that PIP_2_ plays a direct role because PIP_2_ levels in these studies were controlled by overexpressing PIP_2_-modifying proteins. As exemplified for LTP, it remains unclear whether dephosphorylation of PIP_2_ could lead to impairment of LTD. To re-confirm this notion, we induced LTD with low-frequency stimulation (LFS, 1 Hz, 900 pulses) after rapamycin treatment in uninfected and infected hippocampal CA1 neurons. As expected, we observed that the rapamycin treatment did not affect LTD in uninfected hippocampal neurons (Fig. 6a). However, rapamycin treatment blocked LTD in neurons infected with lentivirus containing LDR-T2A-Inp54p, as compared with uninfected control neurons (Fig. 6b). These results indicated that LTD occurring in the CA3-CA1 synapse is inhibited by the rapamycin induced translocation of Inp54p.

To verify that the blockade of LTD induction resulted from the PIP_2_ decrease, we used the catalytically dead mutant LDR-FKBP-Inp54p(D281A)^22^. We tested whether the translocation of FKBP-Inp54p(D281A) affected membrane PIP_2_ levels. However, rapamycin treatment did not result in the translocation of the PH-domain from the plasma membrane to the cytosol, indicating that membrane-bound PIP_2_ was not reduced by Inp54p(D281A) in HEK293T cells (Fig. 6c). LTD was induced normally in CA1 pyramidal neurons expressing FKBP-Inp54p(D281A) in the presence of rapamycin, which does not dephosphorylate PIP_2_ (Fig. 6d,e). These results indicated that the blockade of LTD by the translocation of FKBP-Inp54p results from the decrease of PIP_2_ although we did not directly show the depletion of PIP_2_.

The CID system allows for the control of PIP_2_ levels for several minutes after application of rapamycin (Fig. 1), which can impart rapid temporal control of physiological actions induced by PIP_2_. By applying rapamycin after the induction of LTD, we sought to determine whether membrane PIP_2_ is also required for LTD expression. When rapamycin was perfused after LFS and the ensuing induction of LTD, however, we detected no significant change in the magnitude of LTD in neurons infected or uninfected with lentivirus containing LDR-T2A-Inp54p (Fig. 6f,g). This finding argues against the possible involvement of PIP_2_ in the expression phase of LTD. Collectively, the results of our rapid manipulation of PIP_2_ levels revealed that membrane PIP_2_ is an essential factor in the induction of LTD, likely through the direct action of the phospholipid rather than via the IP_3_ and DAG generated from PIP_2_.

## Discussion

We developed and used CID in hippocampal circuits to resolve the existing disparate observations about direct effects of PIP_2_ on neural and synaptic functions. Contrary to previous findings, this study indicates that PIP_2_ is critically and selectively necessary for LTD induction but not for neuronal excitability, synaptic transmission, LTP expression or LTD expression. In addition, we proved the efficacy of the new method whereby PIP_2_ levels are controlled through immediate translocation of PIP_2_-modulating enzymes to dephosphorylate PIP_2_.

Previous studies have critical limitations in elucidating the direct physiological roles of PIP_2_ in synaptic plasticity in that most relied on genetic manipulation of PIP_2_-metabolizing enzymes in the brain tissues and animal models^9, 14, 15^. The long-term modulation of PIP_2_-modifying enzymes could potentially produce compensatory effects. It might also produce untoward alteration of the other signaling molecules besides membrane-bound PIP_2_, which should compound the physiological or behavioral consequences derived from modulation of PIP_2_ levels. Ectopic expression of the enzymes does not allow a determination of the timeframe during which PIP_2_ can exert its action on each stage of synaptic plasticity. Furthermore, pharmacological modulation of PIP_2_-modifying enzymes has the caveats of potential off-target effects and diffusion time, particularly problematic in brain tissues^25^.

We adopted the CID system and further developed it for a use in brain slices, which allowed us to overcome the aforementioned limitations and resolve conflicting observations for direct roles of PIP_2_ in synaptic transmission and synaptic plasticity in the CA1^14–16^.

Although rapamycin has been shown to block LTP^41^, we ruled out the possibility that our brief treatment with rapamycin at the concentration used (100 nM for 3 min) interferes with synaptic plasticity by observing intact synaptic transmission and normal development of LTP. These data, along with immunohistochemical results, verify the specificity and efficacy of the CID system for temporal control of PIP_2_ level without affecting synaptic features.

We provided substantial but indirect evidences indicating that our CID system could acutely dephosphorylate PIP_2_ in brain slices: 1) rapamycin could result in translocalization of FKBP-Inp54p in CA1 neurons. 2) when the catalytically-dead mutant of Inp54p was used, rapamycin treatment induced the similar redistribution of FKBP-Inp54p but did not affect LTD. Although these findings support the possibility that PIP_2_ is manipulated by CID in brain slices, further studies including quantitative immunostaining for PIP_2_ would be required to ascertain that PIP_2_ is reduced by the used CID method and to analyze changes in the amount of PIP_2_ residing particularly in the brain slices.

We re-assessed at the hippocampal circuit level whether PIP_2_ could directly control neuronal and synaptic features using CID. The acute dephosphorylation of PIP_2_ did not produce a significant alteration in neuronal excitability and synaptic transmission, although it was previously reported that the activity of NMDARs or AMPARs is regulated by appropriate levels of PIP_2_. However, it remains unclear what caused the discrepancies between our and previous studies. Activity of PIP_2_-modifying enzymes, such as PTEN, would regulate individual glutamatergic receptors by altering the channel activity or synaptic distribution of receptors^14^. If this is the case, the previously observed changes in synaptic transmission would have resulted from secondary effects of PIP_2_-modifying enzymes rather than direct PIP_2_ decrease. In aged mice, elevated levels of Ca^2+^ lead to gradual release of the myristoylated alanine-rich C-kinase substrate from the membrane and subsequent loss of PIP_2_, which results in impairment of PLCγ signaling and LTP^40^. However, we failed to detect any changes in LTP at the CA3-CA1 synapse elicited by stimulation of the Schaffer collateral pathway. Importantly, we observed impairment in NMDAR-dependent LTD when PIP_2_ was dephosphorylated selectively at the postsynaptic CA1 neurons by targeted viral infusion. These results clarify previous conflicting findings on the role of PIP_2_ signaling for postsynaptic function and plasticity. Furthermore, our CID enabled us to examine which phase of LTD is dependent upon the basal levels of PIP_2_ by temporally regulating administration of rapamycin, revealing the necessity of PIP_2_ for induction of LTD but not expression. To our knowledge, this is the first study demonstrating that a basal amount of PIP_2_ at the postsynaptic sites is required for LTD induction alone.

The detailed molecular mechanisms underlying reduced LTD induction upon PIP_2_ manipulation remain unclear. One possibility is blockade of AMPAR endocytosis during LTD induction. The binding of clathrin-adaptor, AP-2, to membrane PIP_2_ is essential for triggering clathrin-mediated AMPAR endocytosis induced by LTD^15^. Reduction of membrane PIP_2_ might impede this interaction and decrease surface expression of AMPAR. Further detailed investigations will be required to clarify these issues.

There is mounting evidence for the critical roles of LTD in cognitive functions^42–44^. In fact, NMDAR-dependent LTD is reported to be involved in the development of behavioral flexibility^16, 45^, episodic-like memory^46^, and immediate memory of a novel context^47^. While those previous studies had suggested the causal role of NMDAR-dependent LTD in mental functions, including updating and flexibility of spatial and emotional memory, there have been no effective means to modulate LTD in a time- and circuit-specific fashion. It would be extremely interesting to discover whether and how temporal regulation of NMDAR-dependent LTD has any significant impact on specific types of memory-related behavior. Further investigations using the CID system are underway to elucidate the behavioral and physiological underpinnings of PIP_2_- and LTD-mediated regulation at the circuit and organism levels.

PIP_2_ deficiencies were observed in pathophysiological situations, like brain aging and Alzheimer’s disease (AD)^40, 48^. Although these are likely to be chronic situations, it is possible to examine the chronic effects of PIP_2_ decrease because the CID complex induced by rapamycin is irreversible. Reducing PIP_2_ levels acutely and *in vivo* via CID may provide a relevant model to identify cellular mechanism.

Given the high affinity of rapamycin for its protein-binding partners (*K*_d_ = 0.2 nM for rapamycin–FKBP binding and *K*_d_ = 12 nM for FKBP-rapamycin-FRB)^49^, rapamycin-inducible tools for manipulating signal transduction would be considered irreversible. The irreversibility would preclude extensive usage of this CID for sequential studies to assess physiological roles and molecular mechanisms of PIP_2_ for synaptic plasticity in a time- and phase-dependent manner. Recently, photocleavable rapamycin and the dual CID system were developed for temporal and spatial control of enzymatic activity^50–52^. If reversible rapamycin derivatives are used to construct a novel CID for future investigations, one can gain further molecular insights into the roles of PIP_2_ or phosphoinositide metabolites in synaptic plasticity and animal behavior.

In conclusion, we developed CID system and identified that appropriate levels of PIP_2_ at plasma membrane are critically required for induction of LTD, but not expression. Future studies using this CID will provide ample mechanistic insights into functional roles of PIP_2_ or phosphoinositide metabolites at the neural circuit and potentially organismic levels.

## Methods

### Animal

C57BL/6 mice were housed under a 12-hour light/dark cycle and given *ad libitum* access to food and water. All procedures for animal experiments were approved by the ethical review committee of POSTECH (Pohang University of Science & Technology), Korea and performed in accordance with the relevant guidelines.

### DNA constructs

To generate LDR-T2A-Inp54p, Lyn_11_-targeted FRB (LDR) (plasmid #20147) and CF-Inp54p (plasmid #20155) were obtained from Addgene. As a backbone vector, pCDH-EF1-MCS-T2A-copGFP vector was used. The PCR fragment containing the LDR plus T2A sequence was digested with XbaI and PstI and ligated into the backbone vector between XbaI and PstI. Subsequently, the PCR fragment of CF-Inp54p was digested with PstI and SalI and was inserted into the LDR-T2A sequence containing vector. eCFP was replaced by eGFP amplified from EGFP-N1. In the LDR-T2A-Inp54p construct, Inp54p was superseded by the PCR fragment of Inp54p(D281A) from CF-Inp(D281A) (Addgene, plasmid 20156). PH-GFP (plasmid #21179) and PH-mCherry (plasmid #36075) were purchased from Addgene.

### Cell culture and transfection

HEK293T cells were cultured in Dulbecco’s modified Eagle’s medium (DMEM) supplemented with 10% fetal bovine serum (FBS; Hyclone, Logan, UT) and penicillin–streptomycin (100 U/ml penicillin–100 μg/ml streptomycin) and incubated under an atmosphere of 5% CO_2_ at 37 °C. Transfections were performed using Lipofector Q (AptaBio, South Korea) according to the manufacturer’s instructions. The fluorescence images were obtained 24 h after transfection. HEK293T cells were infected with lentivirus containing LDR-T2A-Inp54p and then 3 days after infection, cells were examined.

### Hippocampal neuron culture, transfection, and immunocytochemistry

Primary hippocampal neurons dissected from the brains of postnatal day 1 (P1) of C57BL/6 mice were plated on poly-L-lysine (Sigma, St. Louis, MO) coated coverslips. Neurons were maintained in neurobasal medium (Invitrogen, Carlsbad, CA) supplemented with B27 (Invitrogen), 5 mM L-glutamine (Sigma) and 1% penicillin–streptomycin (Invitrogen) under an atmosphere of 5% CO_2_ at 37 °C. Neurons were transfected with Calcium Phosphate Transfection Kit (Invitrogen) following the previously described methods^53^. 100 nM rapamycin (LC laboratories, Woburn, MA) were applied for 3 min.

### Virus production

Lentiviral constructs were cotransfected with vesicular stomatitis virus glycoprotein, gag and pol constructs in HEK293T cells by Lipofector Q (AptaBio). 72 h after transfection, viral media were harvested. To remove cellular debris, harvested media were centrifuged at 1,500 rpm for 5 min. Supernatant was filtered through a 0.45 μm syringe filter (Sartorius stedim, Germany) and then concentrated by ultracentrifugation using an SW28 rotor (Beckman Coulter, Brea, CA) at 25,000 rpm for 120 min at 4 °C.

### Stereotaxic injection of lentivirus

C57BL/6 male mice (P21) were anesthetized with ketamine and xylazine mixture by an intraperitoneal injection and placed in a small animal stereotaxic frame (David Kopf instruments, Tujunga, CA). For lentivirus injection, 2 μl total volume was delivered into the dorsal hippocampus CA1 region bilaterally at an average rate of 200 nl/min through a pulled capillary pipette connected to a Nanoject II (Drummond Scientific, Broomall, PA). CA1 injection coordinates were −1.6 mm from Bregma (AP), ± 1.48 mm to the midline (ML) and −1.48 mm ventral to the surface of the skull (DV). Stereotaxic coordinates were adjusted slightly to the weight of each mouse.

### Immunohistochemistry

The brain slices were fixed in 4% paraformaldehyde, embedded in 4% agarose and sliced into 60 µm thick coronal sections by a vibratome (VT1000S, Leica, Germany). Sliced sections were blocked with 6% normal donkey serum (Bethyl Laboratories, Montgomery, TX) and were permeabilized with 0.3% Triton X-100 in phosphate-buffered saline (PBS) at 4 °C for 1 h and then were incubated with rabbit anti-GFP antibody (LF-PA0043, 1:1000, Ab frontier, South Korea) at 4 °C overnight. Brain slices were washed 3 times in PBS and donkey anti-rabbit Alexa 568 conjugated IgG antibody (A10042, 1:500, Invitrogen) was used at 4 °C overnight as a secondary antibody. All brain slices were washed 3 times in PBS, then mounted on the slide glass with UltraCruz mounting medium with DAPI (Santa Cruz, Dallas, TX).

### Image acquisition and analysis

All fluorescence images were acquired with confocal microscopy (Olympus FV1000 or Zeiss LSM 510) using 10×, 40×, 63× objectives. Live cells were maintained at 35 °C during imaging in the live cell chamber (LCI, Korea) and treated with 100 nM rapamycin (LC laboratories) for 3 min. Images were exported from Fluoview viewer (Olympus, Japan) or Zen software (Zeiss, Germany) as TIFF files. Fluorescence intensity was analyzed with Metamorph software (Molecular Devices, Sunnyvale, CA).

### Electrophysiology

Mice were anesthetized with ketamine and xylazine mixture and then perfused transcardially with an ice-cold sucrose slicing solution (20 mM NaCl, 3.5 mM KCl, 1.3 mM MgCl_2_, 1.4 mM NaH_2_PO_4_, 26 mM NaHCO_3_, 11 mM Glucose and 175 mM sucrose). The brain was rapidly removed from the skull and placed in the same solution. Thereafter, coronal slices of the brain containing the hippocampus (400 μM) were made using a vibratome (VT1000s) in the ice-cold sucrose slicing solution, which was equilibrated with a gas mixture of 95% O_2_ and 5% CO_2_. The slices were maintained in oxygenated ACSF (119 mM NaCl, 2.5 mM KCl, 2.5 mM CaCl_2_, 1 mM MgSO_4_, 1.25 mM NaH_2_PO_4_, 26 mM NaHCO_3_ and 10 mM glucose) at room temperature for at least 1 h before recording. Slices were transferred into a recording chamber and perfused with oxygenated ACSF at a rate of 2 ml/min. The brain slices were exposed 100 nM rapamycin (LC laboratories) for 3 min in the recording chamber with ACSF.

Whole-cell patch clamp recordings were made from pyramidal neurons in the CA1. Electrophysiological experiments were performed with Axopatch 200A (Molecular Devices). Data were acquired by pCLAMP 10.4 software (Molecular Devices) and analyzed by Clampfit 10.4 (Molecular Devices). Recordings were under visual guidance using a Leica microscope with both transmitted light and epifluorescence illumination. Uninfected and infected neurons were distinguished base on the presence of fluorescence signal. Patch pipettes (8–10 MΩ) were pulled from borosilicate glass (1B150–4, World Precision Instruments, Sarasota, FL) by an electrode puller (PC-10, Narishige, Japan).

Rheobase and action potentials rate were measured by current clamp mode with a K-gluconate based internal solution (120 mM K-gluconate, 5 mM NaCl, 1 mM MgCl_2_, 0.2 mM EGTA, 10 mM HEPES, 2 mM Mg-ATP and 0.1 mM Na-GTP). For recording of evoked synaptic responses, a stimulating electrode was placed in the CA3 of the hippocampus approximately 0.1 mm from recorded cell bodies in the CA1 of the hippocampus. Recording electrodes were filled with a cesium methane sulfonate based internal solution (130 mM Cesium methane sulfonate, 10 mM HEPES, 0.5 mM EGTA, 8 mM NaCl and 10 mM phosphocreatine, 2 mM Mg-ATP, 0.1 mM Na-GTP and 5 mM QX-314). Bath solution contained 100 μM picrotoxin (PTX) to block GABAα receptor-mediated current. The stimulus intensity was adjusted to evoke synaptic responses that have the amplitudes 35–40% of the maximum EPSC amplitudes. EPSC recording was obtained in the voltage-clamp mode with the cesium methane sulfonate based internal solution. AMPAR-mediated transmission was obtained as the peak amplitude of the EPSC recorded at −70 mV holding potential. NMDAR-mediated transmission was determined as EPSCs recorded at +40 mV holding potential at 50 ms after afferent stimulation. LTP was induced using a pairing protocol by stimulating Schaffer collateral fibers at 2 Hz (80 pulses), while depolarizing the postsynaptic neurons to 0 mV. LTD was induced by low-frequency stimulation (LFS, 900 pulses at 1 Hz), while clamped at −70 mV.

### Statistics

Quantitative data obtained from images are expressed as the mean ± SEM. Statistical analysis of electrophysiology results is expressed as mean ± SEM % of baseline amplitude of EPSCs recorded over at least a 5 min baseline period. The numbers animals used (N) and/or experiments performed (n) are specified in the figure legends. The normality of the data was first tested by the Shapiro-Wilk test. Mann-Whitney U-test or unpaired t-test was used to determine statistical significance between two groups. *P < 0.05; **P < 0.01; or ***P < 0.001.
